# Provision and use of radiotherapy in Europe

**DOI:** 10.1002/1878-0261.12690

**Published:** 2020-05-01

**Authors:** Yolande Lievens, Josep M. Borras, Cai Grau

**Affiliations:** ^1^ Department of Radiation Oncology Ghent University Hospital and Ghent University Belgium; ^2^ Department of Clinical Sciences IDIBELL University of Barcelona Spain; ^3^ Department of Oncology and Danish Center for Particle Therapy Aarhus University Hospital Denmark

**Keywords:** access, availability, equipment, radiotherapy, staffing, utilisation

## Abstract

Radiation therapy is one of the core components of multidisciplinary cancer care. Although ~ 50% of all European cancer patients have an indication for radiotherapy at least once in the course of their disease, more than one out of four cancer patients in Europe do not receive the radiotherapy they need. There are multiple reasons for this underutilisation, with limited availability of the necessary resources – in terms of both trained personnel and equipment – being a major underlying cause of suboptimal access to radiotherapy. Moreover, large variations across European countries are observed, not only in available radiotherapy equipment and personnel per inhabitant or per cancer patient requiring radiotherapy, but also in workload. This variation is in part determined by the country's gross national income. Radiation therapy and technology are advancing quickly; hence, recommendations supporting resource planning and investment should reflect this dynamic environment and account for evolving treatment complexity and fractionation schedules. The forecasted increase in cancer incidence, the rapid introduction of innovative cancer treatments and the more active involvement of patients in the healthcare discussion are all factors that should be taken under consideration. In this continuously changing oncology landscape, reliable data on the actual provision and use of radiotherapy, the optimal evidence‐based demand and the future needs are crucial to inform cancer care planning and address and overcome the current inequalities in access to radiotherapy in Europe.

## Graphical abstract

While about one out of two cancer patients require radiotherapy, more than a quarter of those in need do not receive it. In Europe, large variation exists in radiotherapy provision (figure) and utilisation. In a rapidly changing radiotherapy and oncology environment, reliable data on radiotherapy needs are crucial to inform cancer plans, to address and overcome these current inequalities.

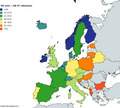

**Abbreviations**
CBB: criterion‐based benchmark
CCORE|: Collaboration for Cancer Outcomes Research and Evaluation
DIRAC: Directory of Radiotherapy Centres
EBEST: epidemiological evidence‐based estimation
ESTRO: European Society for Radiotherapy and Oncology
GNI: gross national income
HERO: Health Economics in Radiation Oncology
MDT: multidisciplinary cancer team
MV: megavoltage
QUARTS: QUAnification of Infrastructure and Staffing Needs

## 1 Introduction

Radiotherapy is an essential part of the multidisciplinary treatment approach for a large number of cancer types. For individual patients, cancer care aims at increasing cure rates, prolonging survival and/or improving health‐related quality of life (Ringborg, 2019). To realise the full impact of innovative interventions on these outcomes and achieve the fundamental aim of a mission‐oriented approach to cancer, the translation of clinical evidence into the healthcare system is crucial and requires alignment over the entire research spectrum, connecting the different components of the cancer research continuum (Celis and Pavalkis, 2017; Fiorino *et al.*, 2020; Lievens, 2017; Ringborg, 2019). There is still some work to be done to attain this goal: in radiotherapy research, for example, clinical and basic sciences by far dominate health services research, the latter representing a mere 2% of the entire radiotherapy research output worldwide (Aggarwal *et al.*, 2018). Several gaps in late translational cancer research have been recognised: translating clinical trial data into real‐life effectiveness and cost‐effectiveness evidence; developing guidelines, including advices for data collection by cancer registries to facilitate outcomes research; and gathering long‐term outcomes and survivorship data.

In addition, the slow and variable implementation of innovative treatment strategies into clinical practice has been described as a major barrier leading to substantial inequalities in cancer care (Ringborg, 2019). This may partly be attributed to the variation in availability of and access to the necessary healthcare resources, which should be addressed (Sullivan *et al.*, 2011). In cancer care, this implies that optimal provision and use of radiotherapy should be guaranteed, if the aim is to achieve the best possible clinical outcomes (Lievens *et al.*, 2019b). If radiotherapy could be deployed so that every cancer patient that requires curative intent radiotherapy is granted access, this would translate into one out of three patients achieving 5‐year local tumour control and 5‐year survival benefits in one out 12 (Hanna *et al.*, 2018). Worldwide, closing the gap to radiotherapy by 2035 would allow to save 1 million lives annually; beyond this curative potential, radiotherapy also plays an important role in alleviating symptoms such as pain, bleeding or obstruction caused by the cancer (Atun *et al.*, 2015).

Proper cancer care planning requires reliable data on the actual situation, on the optimal evidence‐based demand, and on forecasted future needs, of treatments as well as of the related human and capital resources (Borras *et al.*, 2015c; WHO, 2002). The Health Economics in Radiation Oncology project of the European Society for Radiotherapy and Oncology (ESTRO‐HERO) has been developed to generate such evidence for European countries, and focussed on the availability, needs – now and in the future – and costs and reimbursement of radiotherapy in Europe (Borras *et al.*, 2015a; Borras *et al.*, 2016; Borras *et al.*, 2015b; Defourny *et al.*, 2019; Dunscombe *et al.*, 2014; Grau *et al.*, 2014; Lievens *et al.*, 2014; Lievens *et al.*, 2020; Lievens and Grau, 2012). By benchmarking actual data on resource availability and treatments delivered with the optimal evidence‐based needs, an estimation of the gap between optimal and actual use of radiotherapy by country has been made. Such data form the basis for evidence‐based policy decisions that should be framed within a more integrated policy action, such as National Cancer Control Plans (Borras *et al.*, 2015c; WHO, 2002). Examples of similar analyses and subsequent actions carried out at country level – for example in The Netherlands and Denmark – demonstrate the usefulness of such approach to effectively cope with existing availability and access gaps (Overgaard, 2015; Slotman and Vos, 2013).

In this Review article, we first address the needs for and actual use of radiotherapy, and then describe the actual provision of radiotherapy resources in Europe, one of the main factors determining radiotherapy utilisation, to conclude with some policy recommendations that could address the described gaps in use and provision.

## 2 Radiotherapy utilisation: balancing evidence‐based needs with actual use

### 2.1 How many radiation treatments are needed?

In order to evaluate the appropriateness of the actual rate of radiotherapy utilisation in a given cancer population, it is necessary to gain insight into the number of radiation treatments that are needed to provide optimal access to radiotherapy in specific countries or geographic regions, or for separate tumour types. Two methodologically distinct approaches are used.

The criterion‐based benchmark (CBB) is an empirical approach that defines ‘gold‐standard communities', which meet predetermined criteria for optimal access (Mackillop *et al.*, 2015). Thus, the defined optimal rate of radiotherapy utilisation is then used as the benchmark to which all countries or regions should conform. The epidemiological evidence‐based estimation (EBEST), conversely, is a deductive approach derived from evidence‐based radiotherapy indications and epidemiological data, which combined allow estimating an optimal radiotherapy utilisation for each indication in the population of interest. The EBEST approach may be limited to specific tumour types or encompass the full spectrum of cancer diagnoses, the most well‐known example of the latter being the work of the Collaboration for Cancer Outcomes Research and Evaluation (CCORE) group in Australia (Barton *et al.*, 2014; Delaney *et al.*, 2005; Tyldesley *et al.*, 2011).

As it is inherently difficult to define ‘gold‐standard communities’ across highly variable socio‐economic environments and regions, the CBB has limited application in analyses considering multiple countries, whereas the EBEST, due to its comprehensiveness and applicability in various epidemiologic and socio‐economic contexts, has been used in programmes defining optimal radiotherapy utilisation across Europe and worldwide (Atun *et al.*, 2015; Bentzen *et al.*, 2005; Borras *et al.*, 2015a; Borras *et al.*, 2015b). But irrespective of the approach used, some methodologic considerations are required: longer time horizons used for the analysis (e.g. several years to lifelong) typically result in higher estimated needs than shorter time frames (for example, 1 year after diagnosis); while CBB analyses mostly predict lower needs than EBEST studies, variation also occurs amongst different EBEST models, related to the use of more or less restrictive assumptions or different evidence and epidemiology input sources. Moreover, as exercises have been performed in different time frames, in various regions and for a range of tumour types, translating into different treatment standards and cancer population mixes, a range in optimal radiotherapy utilisation figures has been reported in the literature (Atun *et al.*, 2015; Barton *et al.*, 2014; Borras *et al.*, 2015a; Borras *et al.*, 2015b; Lievens *et al.*, 2017).

But regardless of these variations, the calculated radiotherapy needs are converging to a quite consistent average: 50% of all cancer patients have an indication for radiotherapy at least once during the course of their disease, irrespective of the world region evaluated (Atun *et al.*, 2015; Barton *et al.*, 2014; Borras *et al.*, 2015a; Borras *et al.*, 2015b; Lievens *et al.*, 2017).

### 2.2 Does the actual utilisation of radiotherapy in Europe match the needs?

**Fig. 1:**
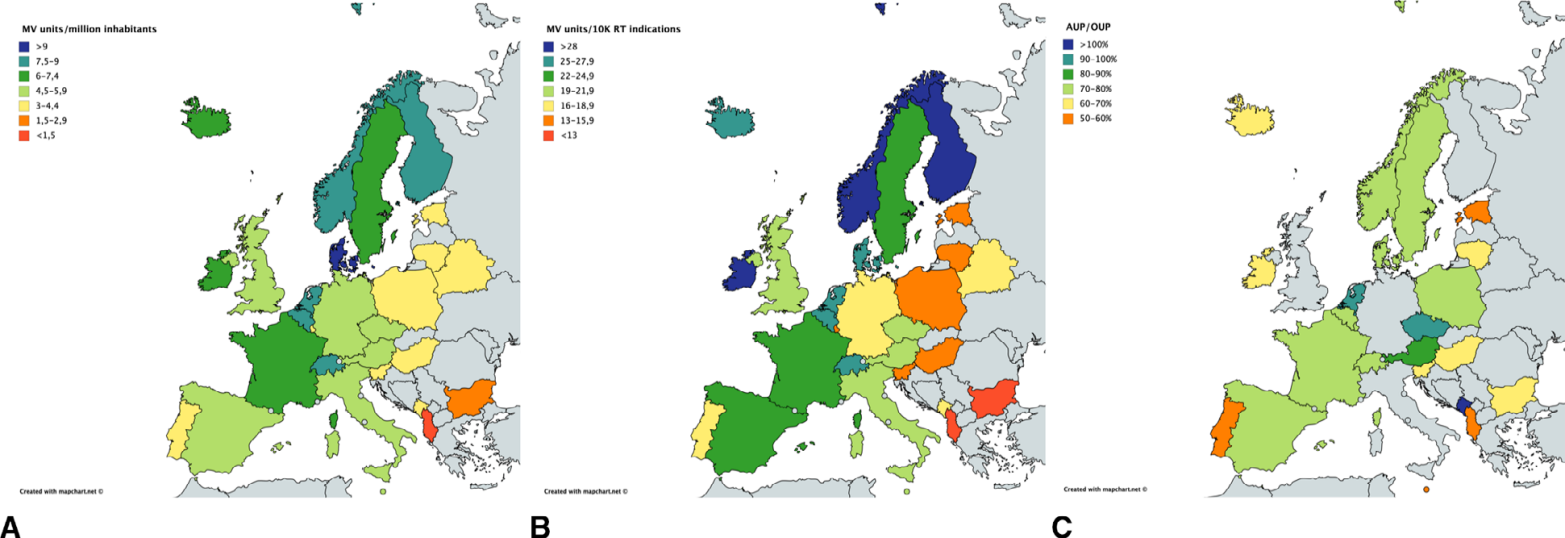
Access to radiotherapy in the European countries, calculated either as the available number of radiotherapy machines per million inhabitants (A) or per 10 000 patients with an indication for radiotherapy (B). The right panel (C) shows the actual utilisation (AUP) of radiotherapy relative to the evidence‐based optimal use (OUP). Based on data from the ESTRO‐HERO project (Borras *et al.*, 2015a; Borras *et al.*, 2015b; Grau *et al.*, 2014).

While the existing evidence suggests that one out of two cancer patients has an indication for radiotherapy, the actual radiotherapy utilisation is much lower. This is especially true for the developing world, where many countries have no radiotherapy equipment available – hence no access – at all (Atun *et al.*, 2015). Underutilisation of radiotherapy is, however, not restricted to low‐ and middle‐income countries. In the pan‐European ESTRO‐HERO project, the actual utilisation was compared to the estimated optimal utilisation at country level, suggesting a large discrepancy amongst European countries. Less than 17% of European countries treat at least 80% of the optimal indications for radiotherapy, and 46% of European countries treat < 70% of the patients with an indication for radiotherapy (Borras *et al.*, 2015b). This utilisation gap is evident even in countries with good access to radiotherapy resources, based on evidence from various European and other high‐income countries (Lievens *et al.*, 2017). Figure 1 shows the heterogeneous distribution of access to radiotherapy in Europe, calculated as the number of radiotherapy machines per million inhabitants (left), or as the number of radiotherapy machines per 10 000 patients with an indication for radiotherapy (middle). Moreover, how the actual utilisation of radiotherapy compares to the evidence‐based optimal utilisation is presented on the right. The fact that the ranking of countries from low to high access varies, depending on the denominator used, illustrates that accounting for the cancer incidence and patient population in a country, rather than just the number of inhabitants, affects the figures of megavoltage (MV) machine availability.

There may be several reasons why patients forego radiation treatment despite evidence‐based recommendations. Patient‐related factors for radiation treatment turndown include high comorbidity and age, low socio‐economic status and education, lack of awareness and information on radiotherapy, and type of primary tumour (Asli *et al.*, 2018; Goossens‐Laan *et al.*, 2014; Lievens *et al.*, 2017; Sundaresan *et al.*, 2017; Vulto *et al.*, 2006a). Moreover, physician‐related bias may apply, as provider characteristics and preferences vary, and specialists tend to recommend their own treatment modalities (Vulto *et al.*, 2005). Geographic factors, as travel distance to a radiotherapy facility and living in rural areas, may also apply (Gabriel *et al.*, 2015).

Insufficient infrastructures, including shortage of human resources and/or equipment, waiting lists or treatment delays, may also impact access to and use of radiotherapy. Evolution of resource availability over time has been shown to impact radiotherapy utilisation in some jurisdictions, not in others (Asli *et al.*, 2014; Barton and Delaney, 2011). Finally, economic factors, including provider costs, and reimbursement barriers affect treatment decisions. It should not be neglected that available methods to assess optimal radiotherapy needs may be partially responsible for the observed gap with actual use, as current models may be overestimating the needs. The relative importance of these factors has not been studied in great detail so far.

In Norway, studies showed that radiotherapy use increased significantly after the implementation of the Norwegian Cancer Plan leading to increased radiotherapy capacity (Asli *et al.*, 2014; Asli *et al.*, 2018). The utilisation reached 42.5% in 2010 but was still lower than the evidence‐based optimum of 53%. For lung and prostate cancer, the actual utilisation was considerably lower than optimal, whereas in breast and rectal cancer the actual use was close to the optimum, even if still suboptimal. For palliative radiotherapy, the Norwegian study found that utilisation was significantly associated with factors such as household income and the availability of a radiotherapy facility at the diagnosing hospital, but even after adjustments for such factors, unexplained geographic variations in palliative radiotherapy utilisation existed. However, expanding resources is not always sufficient for increasing utilisation, as in New South Wales in Australia radiotherapy utilisation remained stable over a decade regardless of the resource investments made; the new facilities could only just keep pace with the increase in new cancer patients with an indication for radiotherapy (Barton and Delaney, 2011).

In Belgium, the actual radiotherapy use and optimal radiotherapy use were compared with the radiotherapy advised during the multidisciplinary cancer team (MDT) conferences in a total of 110 810 cancer patients diagnosed in 2009 and 2010 (Lievens *et al.*, 2017). The results showed that the overall utilisation was 37%, significantly lower than the calculated optimum of 53%, but in line with the advised radiotherapy from the MDT (35%). Large variations by tumour type were observed: for example, in lung and prostate cancer the actual use was considerably lower than the optimal, whereas in breast cancer or head and neck cancer there was a reasonable concordance. In addition, older age was also found to be a barrier to radiotherapy utilisation. A similar negative impact of older age on radiotherapy use was found in the Netherlands. Moreover, whereas the utilisation of primary radiotherapy overall remained stable between 1988 and 2002 in the Netherlands, its use varied considerably for certain tumour types in the same period, reflecting evolving evidence (Vulto *et al.*, 2006b).

The sparse data available thus suggest that the radiotherapy use is lower than the evidence‐based optimum in most European countries. Further analysis of explanations and barriers pertaining to specific countries is needed to better understand the role of radiotherapy in modern multidisciplinary cancer management in various jurisdictions and to plan for future needs. One well‐recognised and important barrier is the lack of resources.

## 3 Radiotherapy resources available across Europe

### 3.1 Provision of equipment

In 2013, the International Atomic Energy Agency published a report on the available radiotherapy equipment and unmet needs in 33 European countries registered in their Directory of Radiotherapy Centres (DIRAC) database (Rosenblatt *et al.*, 2013). In total, 1286 active radiotherapy centres were reported. There was a considerable variation in average number of MV teletherapy machines per radiotherapy centre, ranging between 1.2 and 7.0 across countries. A large variation in department size was also observed, with the largest centres (4–10 MV machines/centre) in Nordic countries, the United Kingdom, the Netherlands and Slovenia, while western and southern European countries had mostly small centres, typically with one or two machines. The number of MV units per million inhabitants also varied considerably (range: 1.3–9.7 MV units per million), with under provision and lower technical capabilities of the equipment seen especially in eastern and south‐eastern European countries. It was concluded that prevailing economic factors affected the available infrastructure, and that the observed fragmentation by itself may entail economic burden, and impact the quality of radiotherapy.

While DIRAC collects data from departments on voluntary basis, the ESTRO‐HERO taskforce collected radiotherapy resource data in Europe at country level, through an 84‐item web‐based survey, which was completed through close interaction between the HERO collaborators and the representatives of the National Societies (NS) for radiation oncology in 40 European countries (Dunscombe *et al.*, 2014; Grau *et al.*, 2014; Lievens *et al.*, 2014). In this most recent dedicated survey on the provision of radiotherapy in Europe, an equally large variation in available equipment and number and size of departments amongst 28 European countries was documented (Grau *et al.*, 2014). The number of MV machines (cobalt, linear accelerators and dedicated stereotactic machines) per million inhabitants ranged from 1.4 to 9.5 (median: 5.3) and the average number of MV machines per department from 0.9 to 8.2 (median: 2.6). In many countries in southern and central‐eastern Europe, there was very limited availability of radiotherapy machines overall and especially of the most updated equipment. The average annual number of radiotherapy courses delivered per MV unit was 419, but again with large variation amongst countries (range: 262–1061). A clear relation to economic strength of the country was noted, a lower gross national income (GNI) per capita predisposing for lower numbers of equipment per inhabitant and for less advanced technologies, thus hampering these countries to adopt the more innovative radiotherapy treatments and techniques.

### 3.2 Provision of personnel

The collection of personnel data at national level, more complex due to the different professional entities and the more quickly changing data over time, was available for 24 countries in the ESTRO‐HERO project (Lievens *et al.*, 2014). It showed an average of 12.8 radiation oncologists per million inhabitants, yet ranging between extremes of 2.5 and 30.9. Similarly, large variability was documented for the other personnel categories, with averages (and ranges) of 7.6 (0–19.7) for physicists, 3.5 (2.7–12.6) for dosimetrists, 26.6 (1.9–78) for radiation therapists and 14.8 (0.4–61) for nurses, per million inhabitants. To account for the fact that radiotherapy professionals fulfil different roles and responsibilities in various countries, further analysis was performed categorising the personnel on the basis of the tasks they perform in the radiotherapy process. This, however, had little impact on the ranges, with 20‐fold variations observed between the highest and lowest staffed countries. In terms of workload, radiation oncologists annually treated 209 courses on average (range: 100–350), while the figures were 303 (85–758) for physicists and dosimetrists combined, and 77 (26–157) for radiation therapists and nurses. Here too, patient throughput is lower in countries with higher GNI/capita, especially for the personnel working in treatment delivery, while the availability of radiation oncologists and medical physicists seems more influenced by other factors, amongst others the tasks they are responsible for. As radiation treatments require highly specialised personnel for treatment preparation, delivery and quality assurance, shortages can only be addressed by training the required staff, which may take years to accomplish.

When observing such important variation in resource availability and workload, one wonders how to explain this, whether it is driven by significantly different needs from different cancer populations to be served, and whether the frequently used population‐based denominator is the most appropriate. In addition, one could question whether the actual situation is supported by the available guidelines by country, and if so how?

### 3.3 Recommendations for radiotherapy staffing and equipment

The QUARTS (RadioTherapy for Cancer: QUAnification of Infrastructure and Staffing Needs) project, conducted by ESTRO more than 15 years ago, provided an overview of the available guidelines for radiotherapy resource needs (Slotman *et al.*, 2005). Based on these, it was suggested that one MV unit could serve 450 patients annually, whereas the personnel needs were defined as one radiation oncologist per 200–250 patients and one physicist per 450–500 patients. Already then, however, it was stressed that these are only crude guidelines, as the actual needs depend on cancer incidence, population mix and treatment strategies, which may differ quite substantially across countries.

Recently, the ESTRO‐HERO project updated these recommendations used to support radiotherapy investments in 29 European countries (Dunscombe *et al.*, 2014). It was quite sobering to see that after more than a decade of clinical, technical and technological evolution in radiotherapy, many countries still used the same guidelines, determined by the same numbers of machine and personnel throughput. Yet, when comparing these recommendations to the available machines and personnel, it was clear that the latter typically outpaced the number set forward by the guidelines, indicating that the actual resources had been adjusted to the needs of innovative and more complex radiation treatments. This underscored the clear need for guidelines that incorporate variations in population and treatment characteristics.

As illustrated in Fig. 1, it is not trivial to consider cancer patients instead of the population to serve when determining radiotherapy resource needs. Different approaches have been reported in the literature.

Directory of Radiotherapy Centres‐based analyses have combined the number of patients who need radiotherapy, estimated at 62.5% of incident cancers (50% of patients for primary radiotherapy, 25% of these for retreatment), with fixed estimates of treatment courses per MV unit or personnel type as described above (Datta *et al.*, 2014; Rosenblatt *et al.*, 2013).

An already more refined approach was used by the ESTRO‐QUARTS project, combining a similar fixed MV machine throughput of 450 courses per year with the epidemiology and clinical evidence on proportions of patients who require radiotherapy for different cancer types, following the CCORE‐EBEST methodology. As such, the differences in MV machines required per million inhabitants were assessed amongst 25 European countries, resulting in an average number of 5.9, yet ranging between 4.0 and 8.1 (Bentzen *et al.*, 2005). Hence, radiotherapy resource needs are indeed, at least partly, driven by cancer incidence and population mix.

Recently, the ESTRO‐HERO project has developed a time‐driven activity‐based costing model for external beam radiotherapy (Defourny *et al.*, 2019). This model not only allows calculating radiotherapy costs, but also allows estimating the quantity of equipment and human resources needed to treat a specific cancer population with radiotherapy. By accounting for the specificities of the population to be treated (proportion of various cancer types, curative vs. palliative intent), combined with complexity and fractionation schedules in clinical use in a country, it estimates the number of radiotherapy resources needed in a more granular manner. In addition, the consequences of changing cancer populations (e.g. due to the instauration of screening programmes) and of varying treatment indications, complexities and fractionation schedules can be assessed, thus forecasting future needs. As such, this model provides an additional and more versatile tool to determine personnel and equipment needs, complementing the more static recommendations of resources needed per number of treatment courses or per population.

## 4 The broader picture of policymaking for the future of radiotherapy provision and use

With increasing pressure on healthcare budgets in most European countries, the radiation oncology community needs to get better insight into the equipment and personnel required to deliver safe, high‐quality and innovative radiotherapy to all cancer patients who need it (Dunscombe *et al.*, 2014). Meanwhile, the large variations observed across Europe in radiotherapy resource provision and use, and the fact that available radiotherapy resources by country, and their utilisation, are partly related to the countries’ GNI instead of actual clinical needs clearly indicates that policy decisions about investment matter.

Three major factors are expected to be drivers of cancer policymaking for the coming years. First, the number of cancer patients is growing, due to an increasing cancer incidence, which is related, amongst others, to the ageing of the population in Europe (Borras *et al.*, 2016; Overgaard, 2015). The fact that aged patients usually have a higher probability of comorbidity stresses the need for a truly multidisciplinary approach to clinical decision‐making (Stairmand *et al.*, 2015). The increasing number of cancer patients amenable to radiotherapy also underscores that recommendations should move away from simple population‐based approaches to models that integrate real patient numbers and evolving practice patterns (Dunscombe *et al.*, 2014).

Second, the continuous introduction of new therapeutic and technological advances imposes various challenges upon the healthcare system. The impact of innovations on the quality of care and clinical outcomes needs to be rapidly assessed, and this is more difficult to accomplish for devices than for pharmaceutical interventions, as the assessment has to be performed prior to or in the early stages of implementation, when access is still limited (Lievens, 2017; Lievens *et al.*, 2019a; Smith *et al.*, 2017). Moreover, healthcare systems need to be made sustainable, also in high‐income regions such as Europe: the growing annual increase in cancer costs is one of the main risks to the future financing of health care (Sullivan *et al.*, 2011). To this end, the combination of new technologies, new drugs and new indications for existing therapies poses a problem without simple answers. Finally, the more active role of patients is changing the traditional patient–physician relationship in the healthcare setting (Leech *et al.*, 2020). The perspective of the patient should be considered when analysing the planning of cancer care.

These factors are deemed to also impact radiotherapy provision and utilisation. While the introduction of novel technologies is recognised as one of the drivers of the increasing healthcare costs, the requirements of health technology assessment for medical devices in Europe lag behind those for drugs (Lievens, 2017; Lievens *et al.*, 2019a). Market entry of new high‐end radiotherapy technologies with higher investment and operational costs, of which particle therapy and magnetic resonance‐guided radiotherapy are typical examples (Grau *et al.*, 2020), urges policymakers to address this issue. Meanwhile, approaches such as managed entry agreements could help to guarantee early patient access to new technologies that have shown consistent and promising data from the initial stages of clinical application (Morel *et al.*, 2013). Real‐world data, including patient‐reported outcome and experience measures, may offer additional insight on the clinical effectiveness of new therapeutic devices (Lievens *et al.*, 2019a).

Moreover, radiation oncology needs to be fully integrated into national cancer plans (Borras *et al.*, 2015c). It is only feasible to address the challenges effectively if we are able to discuss how cancer services should be organised, and how many resources are needed to provide optimal access to all European citizens to high‐quality cancer care. This discussion should be carried out within the framework of a cancer plan, as has been supported by the European Commission and countries through the Joint Actions Against Cancer (https://www.ipaac.eu/).

Cancer policy, like any healthcare policy, involves making decisions that combine high levels of uncertainty and expectations: uncertainty about whether the actual clinical benefit of innovations will match the expectations, and related uncertainty about the actual resources needed to provide access to these innovations. While clinical trials remain the mainstay of evidence generation, they are often more difficult to perform in the context of locoregional cancer strategies (Lievens *et al.*, 2019a). Real‐world data could form the bridge between expectations and clinical efficacy and effectiveness, and provide an additional approach to evidence generation in rapidly evolving environments such as radiation oncology. In parallel, there is also a need to generate evidence on how to organise healthcare provision and delivery, to optimise access for cancer patients in Europe. These new types of evidence can be generated by so‐called late translational research, assessing the dissemination of new technologies, their provision and utilisation, their quality and impact on outcomes (Ringborg, 2019). Only when basing policymaking on this kind of data from across Europe, it would serve the best interests of all European cancer patients.

## 5 Conclusion

While about 50% of all cancer patients have an evidence‐based indication for radiotherapy, more than one out of four cancer patients in Europe do not have access to the radiotherapy they need. Although the reasons underlying this underuse of radiotherapy are multifactorial, the insufficient and highly variable provision of radiotherapy resources, personnel as well as equipment, is one of the main challenges. There is an urgent need to generate more evidence to understand and address the current inequalities in radiotherapy provision and use across Europe, and leverage these data to support multidisciplinary cancer management, cancer planning and policymaking. Only by doing so, we may succeed in providing optimal radiation treatment to every individual cancer patient in Europe, regardless of where he or she lives.

## Conflict of interest

Dr. Lievens reports personal fees from Astra Zeneca and from RaySearch, outside the submitted work. Dr. Grau and Dr. Borras do not report any conflict of interest.
